# Association between urinary 3-phenoxybenzoic acid and body mass index in Korean adults: 1^st^ Korean National Environmental Health Survey

**DOI:** 10.1186/s40557-015-0079-7

**Published:** 2016-01-13

**Authors:** Minsang Yoo, Youn-Hee Lim, Taeshik Kim, Dongwook Lee, Yun-Chul Hong

**Affiliations:** Department of Preventive Medicine, Seoul National University College of Medicine, Seoul, Republic of Korea; Environmental Health Center, Seoul National University College of Medicine, Seoul, Republic of Korea; Institute of Environmental Medicine, Seoul National University Medical Research Center, Seoul, Republic of Korea

**Keywords:** Pyrethroid, 3-phenoxybenzoic acid, Overweight, Obesity, BMI, KNEHS

## Abstract

**Background:**

According to US-EPA report, the use of pyrethrins and pyrethroids has increased during the past decade, and their area of use included not only in agricultural settings, but in commerce, and individual household. It is known that urinary 3-PBA, major metabolite of pyrethroid, have some associations with health effect in nervous and endocrine system, however, there’s no known evidence that urinary 3-PBA have associations with obesity.

**Method:**

We used data of 3671 participants aged above 19 from the Korean National Environmental Health Survey in 2009–2011. In our analysis, multivariate piece-wise regression and logistic regression analysis were used to investigate the association between urinary 3-PBA (3-Phenoxybenzoic Acid) and BMI.

**Result:**

Log-transformed level of urinary 3-PBA had significantly positive association with BMI at the low-level range of exposure (*p* < 0.0001), and opposite associations were observed at the high level exposure (*p* = 0.04) after adjusting covariates. In piece-wise regression analysis, the flexion point that changes direction of the associations was at around 4 ug/g creatinine of urinary 3-PBA. As quintiles based on concentration of urinary 3-PBA increased to Q4, the ORs for prevalence of overweight (BMI ≥ 23 kg/m^2^) were increased, and the OR of Q5 was lower than that of Q4 (OR = 1.810 for Q4; OR = 1.483 for Q5). In the analysis using obesity (BMI ≥ 25 kg/m^2^) as outcome variable, significant associations were observed between obesity and quintiles of 3-PBA, however, there were no differences between the OR of Q5 and that of Q4 (OR = 1.659 for Q4; OR = 1.666 for Q5).

**Conclusion:**

Our analysis suggested that low-level of pyrethroid exposure has positive association with BMI, however, there is an inverse relationship above the urinary 3-PBA level at 4 ug/g creatinine.

**Electronic supplementary material:**

The online version of this article (doi:10.1186/s40557-015-0079-7) contains supplementary material, which is available to authorized users.

## Background

Pesticide, including insecticide, usage is increasing since the past decades. According to U.S. EPA, the amount of global pesticide use in 2007 was about 5.2 billion pounds [1], which reflects a 700 % rise, as compared to 1960s. The pesticide usage in South Korea has also dramatically increased about 20 times during the last 50 years [2], and their field of use includes both agricultural and urban areas.

Pyrethroid, a synthetic chemical insecticide derived from pyrethrins, has been widely used since 1980s because of its effectiveness and low toxicity, as compared to other insecticides such as organophosphorus and carbamic ester compounds. However, similar to other insecticides, pyrethroid insecticides have toxic effects on the nervous system in the body [3, 4]. They act by altering the permeability of sodium ion channels in excited nerve cells [5]. However, distinct from insects, mammals rapidly and enzymatically metabolize pyrethroid insecticides and excrete the metabolites, so that they have lower toxicity to pyrethroid insecticide [6]. Consequently, pyrethroid insecticides in global market comprise more than 30 %, moreover, they are the most widely used agents for indoor pest control [7].

Despite their less toxic effect to human health, recent research revealed that even low-level exposure to pyrethroids also have adverse effects on not only the nervous system but also other systems in humans. There are some reports about immunologic response associated with exposure to pyrethroid [8, 9], behavioral problems in children [10], and neural and mental development of infants who are exposed in the prenatal period [11]. Additionally, some animal studies suggested that the possibility of harmfulness to other systems, including the hepatic system and thyroid [12]. Recently, some animal studies showed body weight change after pyrethroid exposure, as high level exposure was related to decreased body weight or lack of suitably increased body weight [12].

Some studies reported that the relationships between endocrine disrupting chemicals and body weight change might be non-monotonous [13–16]. Especially, the results from some persistent organic pollutants (POPs) showed inverted U-shaped curves with many health outcomes such as type 2 diabetes, dyslipidemia, or weight gain [13, 14, 17]. We aimed to examine the association between pyrethroid exposure and obesity in a national representative cross-sectional study. In addition, we evaluated the differences in the association between pyrethroid exposure and obesity depending on the exposure levels.

## Methods

### Ethics statement

This study was approved by the Institutional Review Board of Seoul National University Hospital (IRB No. 1504-077-665), and when 1st KNEHS was conducted, written informed consent was obtained from all subjects.

### Study participants

We used the data from the 1^st^ Korean National Environmental Health Survey (KNEHS), which was conducted by National Institute of Environmental Research (NIER) from 2009 to 2011. The KNEHS is designed to collect data every 3 years with stratified sample from around 350 survey districts for national representation. The survey included sampling of 2000 people annually. Among the 6,311 participants of 1^st^ KNEHS data, we excluded 2,101 participants who attended the 1^st^ year of survey because of lack of the value of height and weight. Additionally, 539 participants met the exclusion criteria; there were 90 participants who were not actually measured for height and weight, 448 participants with missing values of urinary 3-Phenoxybenzoic Acid (3-PBA), and 1 participant with very extreme value of BMI. After applying the exclusion criteria, the final study population was 3671 participants.

### Variables

12-hour urine and spot urine were collected with sterile specimen cup for urine specimen. The specimen was delivered with opacity in cold storage at 4 °C, and stored below -20 °C if the analysis was delayed. The analytes were separated from the matrix by means of a liquid–liquid extraction, and Clarus 600 Perkin Elmer gas chromatograph equipped with a mass selective detector (Clarus 600 T Mass Spectrometer) was used for the analysis of urinary 3-PBA. In the analysis, the limit of detection was 0.015ug/L, and the target coefficient (R^2^) of calibration curve was same or more than 0.995 for internal quality control. All urinary samples were adjusted with the concentration of urinary creatinine to capture log-normal distribution of urinary 3-PBA levels. Among various demographic characteristics in the survey, we chose to use sex, age, region, current smoking and drinking status, exercise status, education level, use of mosquitocide, and job classification. The definition of overweight and obesity were according to WHO-WPRO criteria: ‘Underweight’ for BMI under 18.5 kg/m^2^, ‘Normal’ for BMI equal or more than 18.5 kg/m^2^ and below 23 kg/m^2^, ‘Overweight’ for BMI equal or more than 23 kg/m^2^ and below 25 kg/m^2^, and ‘Obesity’ for BMI equal or more than 25 kg/m^2^.

### Statistical analysis

Geometric means of urinary 3-PBA were calculated according to various demographic characteristics. Log transformed urinary 3-PBA were used in the analysis due to the right skewed pattern of distribution of urinary 3-PBA. To evaluate the association between urinary 3-PBA and BMI, the figures were plotted using the generalized additive model. Since the direction of association was changed with the increasing level of 3-PBA, piece-wise regression analysis was performed to determine the flexion point with 3 models. Model 1 was applied with simple linear regression analysis, model 2 adjusted age and sex, and model 3 adjusted age, sex and other covariates; Region, current smoking and drinking status, exercise status, education level, use of mosquitocide, and job classification. Flexion point was determined by Akaike information criterion (AIC) in the statistical model, and regression coefficients was calculated in below and above the flexion point, respectively.

Urinary 3-PBA levels were stratified into quintiles by their rank to estimate odds ratios (ORs) and 95 % confidence intervals of prevalence for BMI-related outcomes (‘overweight’ and ‘obesity’). Logistic regression analyses were performed with first quintiles as the reference group.

Since participants of 1^st^ KNEHS were selected with nonrandomized stratified method, statistical analyses were performed using SURVEYREGRESSION and SURVEYLOGISTICS in SAS (Ver 9.3, SAS institute) with proposed sample weights. Generalized additive model of R were used for figures, and *p*-values < 0.05 was considered as statistically significance.

## Results

Table 1 showed the number of participants and geometric means of urinary 3-PBA according to demographic characteristics. The number of total participants was 3671 and the geometric means of creatinine adjusted urinary 3-PBA was 1.83 ug/g. There were 1838 male participants and 1,833 of female participants; and female participants had higher creatinine concentration (2.10 ug/g) than male participants (1.59 ug/g). We observed that older subjects tended to have higher level of urinary 3-PBA, as the concentration of urinary 3-PBA were highest in participants with aged above 60 i.e., 2.78 ug/g creatinine. Participants who lived in ‘Jeju’ had the lowest level, contrastingly, subjects who lived in ‘Honam’, a typical rural area in Korea, had the highest level of urinary 3-PBA i.e., 2.39 ug/g creatinine. There were 868 current smokers and 2193 participants who had a current drinking habit, and they had higher levels. Subjects with low education level had higher 3-PBA level, and the geometric mean was 2.90 ug/g creatinine. In the BMI category, participants who were overweight and obese had higher levels of urinary 3-PBA (1.85 ug/g creatinine for overweight; 2.05 ug/g creatinine for obesity) than participants who had normal BMI (1.63 ug/g creatinine). A number of subjects used mosquitocide only in summer (*N* = 2,798), and subjects using mosquitocide had higher 3-PBA concentration than subjects who did not use mosquitocide (1.90 ug/g creatinine, for subjects with usage only in summer; 1.58 ug/g creatinine for subjects with no using mosquitocide, respectively). Lastly, in job classification, skilled agricultural and fishery workers had higher levels of urinary 3-PBA than subjects who had other job classification, as 2.71 ug/g creatinine.

**Table 1:** General characteristics of study population and geometric means of urinary 3-PBA according to general characteristics

		Concentration of urinary 3-PBA (ug/g Creatinine)	
	*N*	Geometric mean	Geometric SD
Total	3671	1.83	2.71
Sex			
Male	1838	1.59	2.66
Female	1833	2.10	2.70
Age			
19–29	448	0.95	2.41
30–39	696	1.25	2.50
40–49	835	1.69	2.37
50–59	902	2.42	2.46
60≤	790	2.91	2.78
Region			
Metropolitan area and Gangwon	1467	1.68	2.71
Chungchong	458	1.85	2.59
Honam	466	2.39	2.77
Yongnam	1185	1.86	2.72
Jeju	95	1.36	1.99
Current Smoking			
No	2803	1.95	2.73
Yes	868	1.47	2.56
Current Drinking			
No	1478	2.16	2.72
Yes	2193	1.63	2.66
Regular Exercise			
No	2048	1.83	2.76
Yes	1060	1.93	2.65
Irregular exercise	563	1.64	2.60
Education			
Middle school	1113	2.90	2.69
High school	1245	1.80	2.51
College	1313	1.25	2.47
BMI(kg/m^2^)			
< 18.5	100	1.28	2.88
18.5 to < 23	1232	1.63	2.79
23 to < 25	886	1.85	2.68
25 ≤	1453	2.05	2.60
Use of Mosquitocide			
No	826	1.58	2.63
Only summer	2798	1.90	2.73
All year around	36	2.06	2.22
Summer, winter	11	1.83	2.10
Job Classification			
Skilled agricultural and fishery workers	384	2.71	3.05
^a^Indoor worker	1103	1.48	2.54
^b^Outdoor worker	846	1.78	2.44
^c^Others	1338	1.97	2.80

^a^Indoor worker : Legislators, senior officials and managers, Professionals, Clerks, Service workers and shop and market sales workers

^b^Outdoor worker : Craft and related trades workers, Plant and machine operators and assemblers, Elementary occupations, Armed forces

^c^Others : Housewives, Students, Unknown

**Fig. 1:**
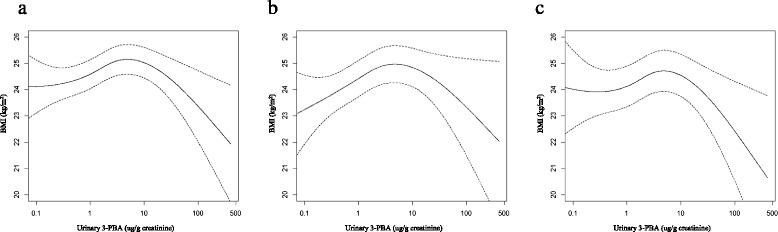
Association between urinary 3-PBA and BMI levels using generalized additive models, among (**a**) the total, (**b**) the male and (**c**) the female participants. The model was adjusted for age, sex, region, current smoking and drinking status, exercise status, education level, use of mosquitocide, and job classification

Figure 1 showed the association between urinary 3-PBA and BMI levels using the generalized additive model. In the range of urinary 3-PBA from 0 to 5 ug/g creatinine, positive associations were observed for the total study population. Contrastingly, in the range of urinary 3-PBA levels above 5 ug/g creatinine, the direction of associations were changed from positive to negative. Similar correlation of increasing BMI with increasing urinary 3-PBA up to a certain point i.e., about 5 ug/g creatinine and decrease after that point were obtained after sex stratification.

Table 2 presented the associations between BMI and urinary 3-PBA obtained from piece-wise regression analysis. From all of 3 models, the flexion point was determined at the value of 1.4 or 1.5 of log-transformed urinary 3-PBA, which is the same as 4.05 or 4.48 ug/g creatinine. There were significant associations between log transformed urinary 3-PBA and BMI in below and above threshold, except at the range above flexion point in model 1(*p* = 0.20); and the direction of associations were changed from positive to negative. In the analysis below the flexion point, the regression coefficient was 0.5830 (*p*-value < 0.0001) in the unadjusted model (Model 1) and 0.4004 (*p*-value < 0.0001) in the fully adjusted model (Model 3). Contrastingly, regression coefficients in the analyses performed in above flexion point was negative, as -0.2137 in model 1. -0.3609 in model 2, and -0.3443 in model 3, respectively.

**Table 2:** Associations between urinary 3-PBA and BMI using piece-wise linear regression analysis

	Flexion point of Log-transformed urinary 3-PBA	Below flexion point		After flexion point	
		Beta-estimate (SE)	*P* value	Beta-estimate (SE)	*P* value
Model 1	1.4	0.5830 (0.0769)	< 0.0001	−0.2137 (0.1679)	0.20
Model 2	1.5	0.4038 (0.0786)	< 0.0001	−0.3609 (0.1792)	0.04
Model 3	1.4	0.4004 (0.0820)	< 0.0001	−0.3443 (0.1679)	0.04

*Model 1 : Crude

Model 2 : Sex, age adjusted

Model 3 : Sex, age, region, current smoking status, current drinking status, regular exercise, education, use of mosquitocide, and job classification adjusted

Table 3 showed the result from logistic regression analysis, and the OR and 95 % CI were calculated for overweight and obesity according to quintiles of 3-PBA. When we used overweight as the outcome variables, almost every quintile in all models had significantly increased prevalence of overweight, as compared to Q1. Furthermore, the ORs increased till Q4, which showed higher OR than that of Q5 (OR = 1.810, 95 % CI 1.329–2.464 for Q4, OR = 1.483, 95 % CI 1.066–2.062 for Q5 in model 3). This result coincided with the result of Table 2. Q4 had the range of 2.257–4.009 ug/g of creatinine, and the maximum of the range was almost the same as the flexion point indicated in Table 2. Analysis using obesity as outcome variable showed similar results as significant positive associations between the prevalence of obesity and almost every quintile in all models. However, there was no difference between the OR of Q4 and that of Q5 (OR = 1.659, 95 % CI 1.211–2.273 for Q4, OR = 1.666, 95 % CI 1.245–2.230 for Q5 in model 3).

**Table 3:** OR and 95 % CI for BMI related outcomes according to quintiles of urinary 3-PBA

< Overweight >						
Quintiles of 3-PBA	Model 1		Model 2		Model 3	
	OR	95 % CI	OR	95 % CI	OR	95 % CI
Q1	Ref		Ref		Ref	
Q2	1.481	1.114–1.970	1.311	0.980–1.755	1.339	0.993–1.806
Q3	1.841	1.395–2.428	1.535	1.143–2.061	1.503	1.113–2.031
Q4	2.256	1.715–2.968	1.813	1.346–2.441	1.810	1.329–2.464
Q5	2.020	1.534–2.662	1.508	1.103–2.060	1.483	1.066–2.062
< Obesity >						
Quintiles of 3-PBA	Model 1		Model 2		Model 3	
	OR	95 % CI	OR	95 % CI	OR	95 % CI
Q1	Ref		Ref		Ref	
Q2	1.384	1.056–1.813	1.263	0.955–1.671	1.277	0.970–1.682
Q3	1.840	1.408–2.405	1.613	1.218–2.137	1.606	1.228–2.100
Q4	1.945	1.454–2.603	1.656	1.201–2.282	1.659	1.211–2.273
Q5	2.100	1.605–2.748	1.715	1.274–2.308	1.666	1.245–2.230

*Range of quintiles: Q1 0.032–0.797, Q2 0.800–1.386, Q3 1.387–2.256, Q4 2.257–4.009, Q5 4.027–261.252

Model 1 : Crude

Model 2 : Sex, age adjusted

Model 3 : Sex, age, region, current smoking status, current drinking status, regular exercise, education, use of mosquitocide, and job classification adjusted

Sensitivity analyses were performed using same method used in Tables 2 and 3 after stratification for sex and participants aged below 60s. (The results were shown in Additional file 1: Tables S1–S5) The flexion points for each sex were 1.6 and 1.2 log-transformed urinary 3-PBA in model 3, respectively. When the analysis performed after exclusion of aged above 60s, we could not find definite flexion point. In the result of the logistic regression analysis with stratification by sex, male participants had highest OR in Q3 (OR = 1.958, 95 % CI 1.268–3.022 in model 3), while that for female participants was observed in Q4 (OR = 1.942, 95 % CI 1.310–2.880 in model 3). Similar result was obtained in obese female. Comparing with Table 3, statistical significance was somewhat weaker, especially in male participants for outcome variable as obesity; however, the overall trend was similar. The same pattern was found in the analysis with aged below 60s for overweight, and the value of peak OR was 1.73 for Q4. However, the result for outcome variable as obesity showed linear increase rather than inverted-U shape.

## Discussion

We determined the association between urinary 3-PBA and obesity in a general adult population by using the nationally representative cross-sectional study of Korea. In conclusion, we observed a positive correlation between urinary 3-PBA and BMI to a certain concentration level, and furthermore, a negative association above a certain threshold.

In this study, we corrected urinary 3-PBA level by using the urinary creatinine level. The geometric mean of creatinine-corrected 3-PBA level in the whole sample was 1.83 ug/g of creatinine. In males, it was 1.59 ug/g and in females, it was 2.10 ug/g. Barr et al. studied 5,046 people from the general population using the NHANES data of the United States from 1999 to 2002 and reported the geometric mean of the urinary 3-PBA concentration as about 0.3 ug/g of creatinine [7]. In a study on German children [18] and on the urban population of Poland [19], the levels were reportedly 0.24 ug/g of creatinine and 0.327 ug/g of creatinine, respectively, showing a marked difference from the results of this study. Meanwhile, among the studies that have targeted Asian countries, Ueyama et al., measured the 3-PBA levels of 535 people from Japan’s middle aged and elderly population [20]. They reported that the geometric mean of urinary 3-PBA concentration was 0.73 ug/g of creatinine, which was higher in males (0.59 ug/g of creatinine) than in females (0.80 ug/g of creatinine). Another group reported a median value of 1.55 ug/g of creatinine in 1,149 pregnant women from an agricultural area of the Province of Jiangsu, China, which was similar to the results from our study [21]. There was little studies about urinary 3-PBA targeted to Korean subjects, but a recent population survey revealed the median concentration of 3-PBA was 1.06 ug/g for male subjects and 1.54 for female subjects [22]. This level of 3-PBA was also higher than other studies conducted in general population from the US or EU countries. Up to now, the reason why Korean has higher urinary 3-PBA level than other countries are in question. Our assumption is that most Koreans were widely exposed to various form of pyrethroid insecticide such as spray and fumigant. Among them, using fumigant insecticide at home is unique form of pyrethroid exposure and has possible common source to general population in Korea. Furthermore, many product including pyrethroid is being advertised as ‘eco-friendly’ product, which has meaning of ‘not toxic’, not as ‘less toxic’.

Recently, ongoing research has focused on the associations between obesity and endocrine disrupting chemicals besides pyrethroids. Most studies for dichlorodiphenyldichloroethylene(DDE) were reported with positive associations with obesity in either cross-sectional or prospective study design [23–26]. While studies about polychlorinated biphenyl(PCB) had inconsistent results of non-significant associations [25–27], positive [28], or inverse associations [29–31]. To overcome these limitations of linearity, some reports argued non-monotonous dose–response (NMDR) relationships between the concentrations of chemicals and body weight [13–15, 17]. That is, inverted U-shaped curve might be more suitable than linearity. One recent prospective study by Lee et al. showed that low dose of p,p’-DDE, p,p’-DDT, and some PCB were associated with BMI in inverted U-shaped relationships [14]. Furthermore, inverted U-shaped responses to exposure of various POPs were observed in dyslipidemia, diabetes and insulin resistance [14, 17]. As known in their name, these chemicals had toxic effect with their persistency in the body. Bisphenol A had a distinct characteristic with these chemicals, as short half-life in humans as less than 6 h similar as pyrethroid. Nevertheless, BPA is well known endocrine disruptor to human and one study reported nonlinear relationships in BMI in the elderly using panel study [32]. In this study, as the concentration of bisphenol A increased, the ORs for overweight showed a corresponding increase at low levels of exposure, however, the response plateaued at high level exposure in total and female subjects. Biological pathway between BPA and obesity were explained as BPA acts through phosphatidylinositol 3-kinase, resulting in accelerated terminal adipocyte differentiation [33], and stimulates triacylglycerol accumulation in mature adipocytes [34]. Some suggested other possible mechanisms that suppression of adiponectin release and a change in hypothalamic action [35, 36]. Some animal studies showed high dose exposure to bisphenol A caused weight loss [37].

Similar to our results, there are studies on animals that show an association between high-exposure to pyrethroid and changes in body weight. According to the toxicological profile for pyrethrins and pyrethroids by ATSDR, a study reported that rats who consumed 250 mg/kg/day of total pyrethrins for 104 weeks lost body weight, and dogs who consumed 12.5 mg/kg/day of fenpropathrin for 3 months had reduced weight gain [12]. Other studies have also reported that intermediate or chronic duration of pyrethrin consumption caused a reduced body weight or body weight gain. However, to our best knowledge, there has been no report on the association between pyrethroids and weight change in human. Only some animal studies indicate that high experimental dosage cause weight loss in animals [38, 39]. However, although direct evidence on influence of low level exposure of pyrethroids on human is not available, we can infer the relation using analogy from some chemicals having similar lipophilic property to 3-PBA. In line with findings of NMDR relationships on bisphenol A, we assume that 3-PBA act like obesogen in low level exposure while behave like toxin in high level exposure. Therefore, we might have significant results due to lipophilic character of pyrethroids in low level exposure and also have inverted U-shaped pattern due to different action depending on the dosage.

There are 2 potential biological mechanisms for pyrethroid mediated effect on body weight. First is affecting the neurological system. The main mechanism is the sodium channels blocking effect of pyrethroids in the nervous system. In animal studies, among nerve tissues, pyrethroid was found to be most highly concentrated in sciatic nerves, then in the order of hypothalamus, frontal cortex, hippocampus, and caudate putamen. Among these, hypothalamus is a well-known central appetite control center [40–42], and various studies describe hippocampus as having an effect on energy intake and body weight regulation [43–45]. Also, Hossain et al. reported that pyrethroid has an effect on the secretion of acetylcholine from hippocampus [46]. Their results suggest the possibilities of weight change on pyrethroid exposure through disturbing the central appetite control centers such as hypothalamus and hippocampus. The second mechanism involves the endocrinologic effects of pyrethroid. Many pesticides act as endocrine disrupting chemicals, and pyrethroid reportedly causes estrogenic responses [47, 48]. As a result, pyrethroid exposure could cause weight gain through stimulating adipocytes and affecting insulin regulation, similar to other substances with estrogenic responses [49, 50].

Our research had several limitations. First, the 1st KNEHS utilizes the data from a cross-sectional study design, which cannot propose a causal relation. Additionally, the half-life of pyrethroids was known as few hours. According to ATSDR, when type II pyrethroids were exposed from oral route, the elimination half-time based on the appearance of metabolites in the urine has been estimated to be between 6 and 13 h. Exposure misclassification may occur due to short biologic half-life of pyrethroid depending on the interval between pyrethroid exposure and time of urine collection, so the result might have a bias that tends toward the null. That is, significant association between pyrethroid exposure and obesity in our analysis might be underestimated. Furthermore, from the point of distribution of pyrethroid in our body after absorption, pyrethroids are rapidly distributed in the adipose tissue, liver, kidneys and the nervous system. So, there was a possibility that participants who had high BMI could get high urinary 3-PBA due to distribution of pyrethroid in the body. Even though some experimental animal studies showed that observed body weight was changed after administration of pyrethroid, our study does not provide clear temporal relationship between pyrethroid exposure and obesity. Further longitudinal design of study for human is required to confirm causal relation. Second, overweight or obesity which was used in our analysis as outcome variables, had highly associated with calorie intake, but our study could not consider calorie intake because of lack of data. Other life style factors such as alcohol drinking or regular exercise status were considered in our analysis, although the information were given as simply ‘Yes’ or ‘No’. Last, this study evaluated pyrethroid exposure with urinary 3-PBA alone. We did not have any information on urinary metabolites of pyrethroid other than 3-PBA, including cis- and trans-DCCA, 4-fluoro-3-phenoxybenzoic acid (4F3PBA). However, urinary 3-PBA is a metabolite of various pyrethroids (permethrin, cypermethrin, deltamethrin, allethrin, resmethrin, fenvalerate, etc.), which enables the assessment of pyrethroid exposure. In spite of these limitations, our study has some strength. First, sample size of our study was larger than any studies for pyrethroid exposure to human in Korea. Second, the 1st KNEHS was designed to have national representation for general population using stratified multistage probability sampling method. However, further longitudinal studies will be required to clarify the causal relationship between pyrethroid exposure and obesity.

## Conclusions

In conclusion, we identified a positive correlation between low level of urinary 3-PBA and BMI, and a negative association above a certain threshold. Pyrethroids are frequently used not only in agricultural areas, but also in the urban areas, hence appropriate management of pyrethroids is required.

## Additional file

Additional file 1:Table S1 Associations between urinary 3-PBA and BMI using piece-wise linear regression analysis for male participants. **Table S2**. Associations between urinary 3-PBA and BMI using piece-wise linear regression analysis for female participants. **Table S3**. OR and 95 % CI for BMI related outcomes according to quintiles of urinary 3-PBA for male participants. **Table S4**. OR and 95 % CI for BMI related outcomes according to quintiles of urinary 3-PBA for female participants. **Table S5**. OR and 95 % CI for BMI related outcomes according to quintiles of urinary 3-PBA after exclusion of aged above 60s. (DOCX 28.4 kb)
