# Supplementary material for: Association between urinary 3-phenoxybenzoic acid and body mass index in Korean adults: 1st Korean National Environmental Health Survey
Source: Ann Occup Environ Med. 2016 Jan 13;28:2. doi: 10.1186/s40557-015-0079-7 (PMC4711175; doi:10.1186/s40557-015-0079-7)
Supplement: Additional file 1:Table S1 — Associations between urinary 3-PBA and BMI using piece-wise linear regression analysis for male participants. Table S2. Associations between urinary 3-PBA and BMI using piece-wise linear regression analysis for female participants. Table S3. OR and 95 % CI for BMI related outcomes according to quintiles of urinary 3-PBA for male participants. Table S4. OR and 95 % CI for BMI related outcomes according to quintiles of urinary 3-PBA for female participants. Table S5. OR and 95 % CI for BMI related outcomes according to quintiles of urinary 3-PBA after exclusion of aged above 60s. (DOCX 28.4 kb) [file 40557_2015_79_MOESM1_ESM.docx]

| **Supplementary Table 1. Associations between urinary 3-PBA and BMI using piece-wise linear regression analysis for male participants.** | | | | | | |
| --- | --- | --- | --- | --- | --- | --- |
|  | **Flexion point of Log-transformed urinary 3-PBA** | **Below flexion point** | | **After flexion point** | | |
|  |  | **Beta-estimate (SE)** | ***P* value** | **Beta-estimate (SE)** | ***P* value** |  |
| **Model 1** | 1.5 | 0.3915 (0.1630) | 0.01 | -0.7613 (0.4485) | 0.09 |  |
| **Model 2** | 1.6 | 0.3123 (0.1761) | 0.07 | -0.6298 (0.4411) | 0.15 |  |
| **Model 3** | 1.6 | 0.3457 (0.1760) | 0.05 | -0.5720 (0.4279) | 0.18 |  |
| * Model 1 : Crude | | | | | | |
| Model 2 : Age adjusted | | | | | | |
| Model 3 : Age, region, current smoking status, current drinking status, regular exercise, education, use of mosquitocide, and job classification adjusted | | | | | | |

| **Supplementary Table 2. Associations between urinary 3-PBA and BMI using piece-wise linear regression analysis for female participants.** | | | | | | |
| --- | --- | --- | --- | --- | --- | --- |
|  | **Flexion point of Log-transformed urinary 3-PBA** | **Below flexion point** | | **After flexion point** | | |
|  |  | **Beta-estimate (SE)** | ***P* value** | **Beta-estimate (SE)** | ***P* value** |  |
| **Model 1** | 1.2 | 0.6518 (0.1658) | < 0.0001 | -0.3237 (0.3393) | 0.71 |  |
| **Model 2** | 1.2 | 0.5607 (0.1753) | 0.01 | -0.4598 (0.3475) | 0.18 |  |
| **Model 3** | 1.2 | 0.4627 (0.1816) | 0.01 | -0.5327 (0.3494) | 0.12 |  |
| * Model 1 : Crude | | | | | | |
| Model 2 : Age adjusted | | | | | | |
| Model 3 : Age, region, current smoking status, current drinking status, regular exercise, education, use of mosquitocide, and job classification adjusted | | | | | | |

| **Supplementary Table 3. OR and 95% CI for BMI related outcomes according to quintiles of urinary 3-PBA for male participants.** | | | | | | |
| --- | --- | --- | --- | --- | --- | --- |
| **< Overweight >** | | | | | | |
| **Quintiles of 3-PBA** | **Model 1** |  | **Model 2** |  | **Model 3** |  |
|  | **OR** | **95% CI** | **OR** | **95% CI** | **OR** | **95% CI** |
| **Q1** | Ref |  | Ref |  | Ref |  |
| **Q2** | 1.295 | 0.879-1.908 | 1.209 | 0.820-1.781 | 1.244 | 0.815-1.897 |
| **Q3** | 2.072 | 1.357-3.163 | 1.839 | 1.202-2.814 | 1.958 | 1.268-3.022 |
| **Q4** | 1.881 | 1.202-2.944 | 1.614 | 1.007-2.586 | 1.673 | 1.016-2.756 |
| **Q5** | 1.881 | 1.267-2.792 | 1.523 | 0.987-2.349 | 1.690 | 1.074-2.660 |
| **< Obesity >** | | | | | | |
| **Quintiles of 3-PBA** | **Model 1** |  | **Model 2** |  | **Model 3** |  |
|  | **OR** | **95% CI** | **OR** | **95% CI** | **OR** | **95% CI** |
| **Q1** | Ref |  | Ref |  | Ref |  |
| **Q2** | 1.098 | 0.725-1.602 | 1.081 | 0.713-1.639 | 1.088 | 0.717-1.650 |
| **Q3** | 1.932 | 1.309-2.852 | 1.881 | 1.265-2.799 | 2.044 | 1.373-3.043 |
| **Q4** | 1.298 | 0.845-1.993 | 1.255 | 0.800-1.968 | 1.307 | 0.820-2.083 |
| **Q5** | 1.858 | 1.248-2.767 | 1.775 | 1.163-2.708 | 1.933 | 1.254-2.981 |
| * Range of quintiles: Q1 0.040-0.711, Q2 0.714-1.186, Q3 1.187-1.914, Q4 1.917-3.404, Q5 3.412-261.252  Model 1 : Crude | | | | | | |
| Model 2 : Age adjusted | | | | | | |
| Model 3 : Age, region, current smoking status, current drinking status, regular exercise, education, use of mosquitocide, and job classification adjusted | | | | | | |

| **Supplementary Table 4. OR and 95% CI for BMI related outcomes according to quintiles of urinary 3-PBA for female participants.** | | | | | | |
| --- | --- | --- | --- | --- | --- | --- |
| **< Overweight >** | | | | | | |
| **Quintiles of 3-PBA** | **Model 1** |  | **Model 2** |  | **Model 3** |  |
|  | **OR** | **95% CI** | **OR** | **95% CI** | **OR** | **95% CI** |
| **Q1** | Ref |  | Ref |  | Ref |  |
| **Q2** | 1.758 | 1.255-2.461 | 1.352 | 0.949-1.925 | 1.293 | 0.895-1.869 |
| **Q3** | 1.998 | 1.389-2.873 | 1.291 | 0.881-1.893 | 1.177 | 0.784-1.768 |
| **Q4** | 3.561 | 2.503-5.065 | 2.108 | 1.448-3.069 | 1.942 | 1.310-2.880 |
| **Q5** | 2.825 | 1.952-4.088 | 1.391 | 0.910-2.127 | 1.205 | 0.774-1.877 |
| **< Obesity >** | | | | | | |
| **Quintiles of 3-PBA** | **Model 1** |  | **Model 2** |  | **Model 3** |  |
|  | **OR** | **95% CI** | **OR** | **95% CI** | **OR** | **95% CI** |
| **Q1** | Ref |  | Ref |  | Ref |  |
| **Q2** | 1.608 | 1.110-2.330 | 1.261 | 0.870-1.828 | 1.201 | 0.819-1.761 |
| **Q3** | 2.227 | 1.515-3.274 | 1.521 | 1.031-2.244 | 1.382 | 0.923-2.069 |
| **Q4** | 3.455 | 2.339-5.105 | 2.185 | 1.458-3.275 | 1.973 | 1.311-2.971 |
| **Q5** | 2.890 | 2.051-4.072 | 1.548 | 1.057 | 1.311 | 0.887-1.937 |
| * Range of quintiles: Q1 0.032-0.939, Q2 0.944-1.598, Q3 1.602-2.632, Q4 2.634-4.650, Q5 4.651-202.178  Model 1 : Crude | | | | | | |
| Model 2 : Age adjusted | | | | | | |
| Model 3 : Age, region, current smoking status, current drinking status, regular exercise, education, use of mosquitocide, and job classification adjusted | | | | | | |

| **Supplementary Table 5. OR and 95% CI for BMI related outcomes according to quintiles of urinary 3-PBA after exclusion of aged above 60s.** | | | | | | |
| --- | --- | --- | --- | --- | --- | --- |
| **< Overweight >** | | | | | | |
| **Quintiles of 3-PBA** | **Model 1** |  | **Model 2** |  | **Model 3** |  |
|  | **OR** | **95% CI** | **OR** | **95% CI** | **OR** | **95% CI** |
| **Q1** | Ref |  | Ref |  | Ref |  |
| **Q2** | 1.498 | 1.053-2.130 | 1.327 | 0.925-1.903 | 1.381 | 0.955-1.997 |
| **Q3** | 1.674 | 1.235-2.269 | 1.416 | 1.024-1.957 | 1.413 | 1.017-1.964 |
| **Q4** | 2.060 | 1.459-2.909 | 1.714 | 1.178-2.494 | 1.730 | 1.172-2.554 |
| **Q5** | 2.094 | 1.546-2.835 | 1.648 | 1.189-2.285 | 1.613 | 1.142-2.279 |
| **< Obesity >** | | | | | | |
| **Quintiles of 3-PBA** | **Model 1** |  | **Model 2** |  | **Model 3** |  |
|  | **OR** | **95% CI** | **OR** | **95% CI** | **OR** | **95% CI** |
| **Q1** | Ref |  | Ref |  | Ref |  |
| **Q2** | 1.260 | 0.902-1.760 | 1.168 | 0.834-1.637 | 1.206 | 0.861-1.690 |
| **Q3** | 1.622 | 1.191-2.209 | 1.501 | 1.096-2.055 | 1.516 | 1.127-2.040 |
| **Q4** | 1.775 | 1.253-2.516 | 1.650 | 1.129-2.411 | 1.663 | 1.133-2.441 |
| **Q5** | 2.126 | 1.573-2.874 | 1.956 | 1.447-2.644 | 1.948 | 1.442-2.633 |
| * Range of quintiles: Q1 0.032-0.722, Q2 0.723-1.238, Q3 1.238-1.994, Q4 1.995-3.446, Q5 3.446-80.024  Model 1 : Crude | | | | | | |
| Model 2 : Sex, age adjusted | | | | | | |
| Model 3 : Sex, age, region, current smoking status, current drinking status, regular exercise, education, use of mosquitocide, and job classification adjusted | | | | | | |
